# Leadership and culture change to advance innovation in women’s health

**DOI:** 10.1136/bmj-2025-085994

**Published:** 2025-10-10

**Authors:** Sapna Kedia, Sylvia Kiwuwa-Muyingo, Radhika Uppal, Julius Kirimi Sindi, Evelyn Gitau

**Affiliations:** 1 International Center for Research on Women (ICRW), Asia, New Delhi, India; 2 African Population and Health Research Center, Nairobi, Kenya; 3 Science for Africa Foundation (SFA Foundation), Nairobi, Kenya

## Abstract

**Sapna Kedia and colleagues** argue that equitable leadership, supported by training and systemic reform and rooted in marginalised groups’ experiences and community insight, can transform the innovation cycle, from research and development to delivery, making health technologies more inclusive and impactful

## Introduction

When women are excluded from leadership, women’s health innovation is incomplete and inequitable and will continue to fall short. Women, especially those from low and middle income countries, remain under-represented in senior positions across global health institutions, research leadership, and innovation funding, even when the research and innovation concerns their own communities.^1^ Grant making mechanisms often favour institutions with extensive administrative infrastructure and previous funding experience, limiting access for women from under-resourced settings.^2^ Furthermore, institutional cultures reflect gendered expectations around leadership style, mobility, and availability. Historical systems that reward linear and uninterrupted career trajectories are misaligned with the realities of many women researchers, who may be balancing care giving responsibilities or facing other gendered constraints.

The absence of women, particularly those from low and middle income countries, in research and innovation leadership has profound implications not only for equity but also for the effectiveness and relevance of health innovation and its impact on health outcomes. Clinical and medical research remains predominantly led by men. For example, many cardiovascular drug trials in South Asia have primarily enrolled men,^3^ resulting in diagnostic and treatment protocols that are based on male physiology and inadequately tested for effectiveness in women. In addition, health conditions that uniquely or disproportionately affect women, such as endometriosis and maternal morbidity, continue to be under-researched and underfunded.^4^ Beyond this, a broader spectrum of women’s health problems across the life course is often rendered invisible owing to the persistent focus on reproductive health.^5^

In this article, we argue that equitable leadership in health research and innovation is not only a matter of rights but also a strategic lever to drive more inclusive, effective, and scalable innovation in women’s health. It requires the embedding of sex and gender considerations into education and training, the dismantling of institutional and systemic barriers, and the protection of rights. We outline a framework for equitable leadership across the innovation cycle from research and development to delivery, and we highlight case studies that show how women leaders are reshaping health innovation to better serve communities worldwide. This article is part of the BMJ’s Gates Collection on Women’s Health Innovation, which aims to identify pathways for advancing innovation in women’s health that are not only technologically cutting edge but also inclusive and equitable.

## Pathways to equitable leadership in women’s health innovation

In women’s health, innovation refers to new solutions that meet unmet gender specific needs across multiple domains. These span institutional innovations, such as gender responsive research and regulatory policies that create frameworks that better serve women^6^; technological innovations including novel drugs, diagnostics, devices, and digital health tools tailored for women^7^; business and market innovations, such as the growing “fem tech” sector and women focused service models that expand access to care^8^; and social innovations including community education and empowerment programmes that tackle the social determinants of health.

Health innovations typically progress through a series of stages, from early research and development and proof of concept to clinical testing, financing, regulatory approval, and ultimately delivery and scale-up. Each phase involves a diverse set of actors—funders, researchers/entrepreneurs, regulators, implementers, and patient communities. Equitable leadership at each stage matters so that the needs and knowledge of marginalised groups including women and gender diverse individuals are considered and their health outcomes are positively affected. However, systemic barriers to advancing equitable leadership persist. Women and gender diverse innovators remain under-represented in leadership and policy making roles, and their ventures remain underfunded. Women’s health still receives only a tiny fraction of funding—5% of global health research and development funding is directed to women’s health.^9^ Regulatory processes have neglected to mandate adequate participation of women and gender diverse populations in clinical trials, and male dominated investor networks often fail to recognise women centred innovations as viable markets

Equal representation and leadership in early research and development can shift research agendas towards the needs of the underserved communities. For instance, women researchers have helped to shift HIV prevention strategies in Africa by studying the dapivirine vaginal ring, which reduced HIV risk by 30% among women at high risk.^10^ In financing, investors that apply a gender equitable lens and have inclusive boards are more likely to direct resources to capital into women’s health. Inclusive regulators and policy makers can set equitable standards and requirements and corresponding accountability mechanisms for research and development, by mandating the inclusion of women and gender diverse people in clinical trials. Finally, inclusive leaders in implementation can ensure that new products and services reach marginalised groups effectively, thereby generating more equitable health outcomes.

**Fig 1:**
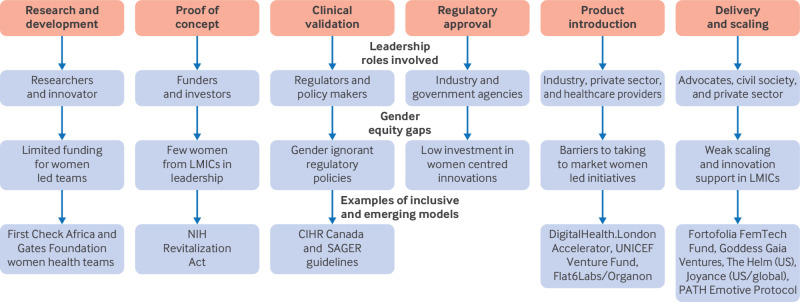
Pathways to understanding equitable leadership in women’s health innovation at each stage of the innovation pathway. CIHR=Canadian Institutes of Health Research; LMIC=low and middle income country; NIH=National Institutes of Health

The conceptual framework below maps stages of the health innovation lifecycle and identifies where gaps in leadership persist and where inclusive models are beginning to emerge (fig 1). It illustrates the leadership roles involved at each phase (for example, funders, innovators, regulators, implementers) and identifies key bottlenecks for women, particularly those in low and middle income countries, across the pipeline. Examples of transformative approaches, such as gender responsive funding platforms, inclusive regulatory reforms, and feminist accelerator models, show where progress is underway.

At each stage of the pipeline, leadership roles are pivotal, yet women, particularly in low and middle income countries, are often missing. Gaps in early stages cascade downstream, limiting the diversity and relevance of solutions that reach scale.

*Early research and development and proof of concept*—Funders (governments, philanthropies, venture capitalists) and researchers set the agenda in this phase. Funding inequities and exclusion prevent many women’s ideas from advancing. Globally, only 2.3% of venture capital goes to all-women founding teams,^11^ with even less directed to health innovators based in low and middle income countries. In health technology, even less flows to women led projects, with less than 2% of all venture capital being invested in women’s companies and just 1.4% going to fem tech and only 4% of health research and development funding targeting women’s health.^12^ This “funding gap” prevents many women’s ideas ever reaching proof of concept. Promising models are emerging—for example, gender lens funds such as Sweef Capital, a women run and women focused equity investment firm in South East Asia, and grants such as women’s health portfolios at major foundations that explicitly target women led ventures.

*Clinical validation and regulation*—Researchers, clinical investigators, and regulatory bodies (ethics boards, drug/device agencies) take the lead in this phase. Women continue to be under-represented in trial design, review processes, and regulatory bodies. Despite the National Institutes of Health Revitalization Act of 1993 mandating inclusion of women in US funded clinical trials, gender bias in trial protocols and decision making remains a global challenge.^13^ The Canadian Institutes of Health Research^14^ and the SAGER guidelines have since advanced these reforms by embedding sex and gender based analysis into research design and review processes, aiming to redress historic under-representation and improve research rigour.

*Product introduction and commercialisation*—Entrepreneurs, start-ups, and industry funders drive this stage. Women led health ventures repeatedly encounter “the funding cliff”; even with viable products, raising growth capital is hard. The structural bias is stark: despite female founders making up a large share of women’s health start-ups, they receive only a tiny fraction of investment.^15^ Design biases also occur; many innovations overlook women’s lived needs because development teams lack diverse leadership. On the positive side, several equity focused funds and incubators have appeared (for example, fem tech venture funds such as Portfolia, Goddess Gaia, and the Helm), mainly in high income countries so far. Expanding similar mechanisms in low and middle income countries is a growing priority to ensure that women innovators get needed capital and mentorship.^16^

*Delivery and scale-up*—Health ministries, non-governmental organisations, procurement agencies, and networks implement products at scale. Here, women led solutions can falter if not embraced by health systems. Barriers include gender ignorant procurement (small, women run firms may lack access) and lack of women decision makers in health programmes. Some inclusive initiatives tackle this: for instance, gender inclusive start-up networks and innovation hubs (for example, UNICEF’s Venture Fund, DigitalHealth.London’s fem tech accelerators, and Flat6Labs/Organon) are beginning to connect innovators with public systems, sometimes embedding gender equity into their criteria. Innovative initiatives based in low and middle income countries, such as Kenya’s HealthTech Hub Africa, are piloting integrated approaches to connect innovators directly with government health systems, potentially serving as scalable models for inclusive delivery.^17^ ^18^

Taken together, these gaps illustrate why equitable leadership matters—why who leads shapes which problems are prioritised, how solutions are designed, and whether innovations reach the people who need them the most. Building equity into leadership at every stage of the pipeline is therefore essential for impactful women’s health innovation.

## Some emerging models

A growing number of women led innovations are beginning to reshape the health innovation landscape. Table 1 highlights three diverse cases: Kasha, a digital health platform expanding access to women’s health products in East Africa; CognitiveCare, an artificial intelligence powered maternal health risk prediction tool operating in India and East Africa; and Chava, a WhatsApp based sexual and reproductive health education initiative for young Lainas. Each illustrates how women’s leadership interacts with systemic enablers and barriers across the innovation ecosystem and reveals system level lessons about advancing gender equity in health research and innovation.

**Table 1:** Some illustrative examples of women led innovation and their interaction with the framework

Innovation	Barrier	Enabler	Key lessons
Kasha—a digital health platform delivering women’s health products in East Africa^19^	Faced bias from male investors and complex cross boarder regulations on pharmaceutical licences	Supportive national policy environment in Rwanda; early backing from female investors; growing investor recognition of women’s health opportunities; received large scale funds from venture capital and development and finance institutions	Women’s leadership rooted in lived experience helped to align product with market need and diverse financing, and community delivery created locally suitable and sustainable growth
CognitiveCare—AI powered maternal health risk prediction tool^20^	Limited venture capital interest in maternal health; need for validation across diverse health systems; concerns about AI replacing clinicians	Collaboration with established health systems showed scalability in low resource settings and designed to support not replace healthcare workers, easing concerns about AI in clinical care	Women leaders with lived maternal health experiences bought often overlooked critical insights; diverse leadership shaped inclusive innovation; grant funding was vital where venture capital investment is limited; south-north institutional collaborations enabled context specific solutions; validation through research played crucial role in buy-in
Chava—WhatsApp based comprehensive sexual health education for young women^21^	Restrictive policies on sexual and reproductive health information and funding gaps owing to misalignment with venture capital metrics	Inclusive use of technology bypassed formal barriers and enabled easy access; research partnerships supported validation	Support by accelerators that back women focused health technology enabled progress; prioritisation of social impact over commercial returns led to alignment with impact investment models; technology design tailored to real access challenges

AI=artificial intelligence.

In each of these cases, equitable leadership shaped innovation priorities and approaches. Kasha’s focus on stigma reduction and community level delivery, CollectiveCare’s emphasis on early risk detection on the basis of personal experience, and Chava’s commitment to comprehensive education all reflect perspectives often absent from male led health technology development. Women’s leadership at early stages of innovation significantly influenced problem identification, solution design, and implementation approaches across these initiatives. They also reflect the continued importance of diverse funding mechanisms, from venture capital to grants to impact investment, in supporting women led health innovations. Thirdly, the cases show that successful navigation of the innovation ecosystem requires strategic adaptation to local regulatory, cultural, and market conditions, with women leaders often demonstrating skill in developing locally responsive approaches while maintaining scaling potential. Finally, these cases illustrate that women led health innovations often tackle gaps overlooked by traditional health system and technology approaches, suggesting that increasing women’s representation throughout the innovation ecosystem could significantly expand the range and effectiveness of health technologies available to underserved populations globally.

## Conclusions and recommendations

Our framework and case studies show that advancing equitable leadership in women’s health innovation requires targeted interventions that reach beyond individual opportunities and tackle systemic barriers across the innovation ecosystem. Funding organisations need to implement gender responsive investment targets. Action is needed across four domains: funding, institutions, policy, and workforce development.

Funding organisations must implement gender responsive investment strategies. These include setting clear targets for directing capital towards women led ventures and women’s health technologies, mandating gender diversity in grant review panels and requiring unconscious bias training for reviewers. Blended finance mechanisms that reduce investment risk for women led health companies are needed, particularly in low and middle income country markets where commercial returns may be limited but health impact is substantial. Sharing best practices across funding platforms can accelerate adoption of these approaches

Academic and research institutions must embed sex and gender considerations into the education and training of the current and future research workforce. Supportive measures such as parental leave extensions for research timelines and childcare provision during fieldwork are essential to enable women’s participation. Advancement and promotion systems must be restructured to value collaborative, community based research and to recognise non-linear career trajectories. Such institutions should establish regional women’s health innovation hubs in sub-Saharan Africa, South Asia, and Latin America that provide technical assistance for intellectual property protection and technology transfer.

Policy makers and regulators must mandate gender responsive regulatory frameworks that require sex and gender considerations at all stages of research and innovation. Regulatory agencies should set requirements for inclusive trial designs and diverse leadership in review processes. Fast track approval pathways should be introduced for innovations tackling neglected women’s health conditions. National health policies and procurement systems should include targets for women’s participation in leadership and ensure that women led enterprises can compete for contracts. Legal and policy protections against discrimination in research and innovation spaces are critical for safeguarding women’s participation.

Workforce and implementation systems must ensure that women are not only leaders of innovation but also active participants in its delivery. Training community health workers on women specific health technologies, establishing demonstration sites in different regions, and building cross-sectoral partnerships can support scale-up.

Finally, global accountability mechanisms should require all publicly funded health innovation institutions to publish annual gender equity reports building on models such as the Global Health 50/50 public scorecards. Public ranking of institutional performance can create incentives for change when implemented consistently.^22^ Equitable health innovation demands equitable leadership at every link of the chain.

**Key messages**

- The absence of women, particularly those from low and middle income countries, in research and innovation leadership undermines the equity, effectiveness, and relevance of women’s health solutions
- Equitable leadership in health innovation requires that marginalised groups including women and gender diverse individuals are actively involved across the innovation cycle and that their specific needs are supported by institutional reforms
- Equitable leadership is not only about who holds power but about how decisions are made; integrating diverse experiences and community rooted perspectives at every stage of the innovation cycle results in more inclusive, responsive, and impactful health solutions
- Ensuring equitable leadership in health innovation requires coordinated action from policy makers, funder institutions, and health systems, as well as global accountability mechanisms that track equity

## Notes

Contributors and sources: SK conceived and led the development of this article, drawing on 15 years of experience in gender equality, sexual and reproductive health and rights, women’s leadership, and health research, policy advocacy, and programme implementation in South Asia. SK, SKM, and RU co-led the conceptual framing and drafting of the article, informed by their collective expertise in advocating for gender responsive systems and strengthening women’s leadership in health research across low and middle income countries in Asia, in Africa, and globally. JKS and EG contributed their expertise on case studies on health innovations and related recommendations for advancing equitable leadership. The paper draws on evidence and insights from published research, programmatic evaluations, and institutional reports. SK is the guarantor.

AI use: Artificial intelligence has been used for identifying relevant resources, which helped in the development of an outline for the paper, and for language editing.

Provenance and peer review: Commissioned; externally peer reviewed.

This article is part of a collection developed in partnership with the Gates Foundation. Open access fees were funded by the Gates Foundation. *The BMJ* commissioned, peer reviewed, edited, and made the decision to publish. Smruti Patel and Jocalyn Clark were the lead editors for *The BMJ*.
